# Reversal of the Δ*degP* Phenotypes by a Novel *rpoE* Allele of *Escherichia coli*

**DOI:** 10.1371/journal.pone.0033979

**Published:** 2012-03-16

**Authors:** Owen P. Leiser, Emily S. Charlson, Henri Gerken, Rajeev Misra

**Affiliations:** 1 The Microbial Genetics and Genomics Center, Northern Arizona University, Flagstaff, Arizona, United States of America; 2 Department of Microbiology, University of Pennsylvania School of Medicine, Philadelphia, Pennsylvania, United States of America; 3 School of Life Sciences, Arizona State University, Tempe, Arizona, United States of America; Baylor College of Medicine, United States of America

## Abstract

RseA sequesters RpoE (σ^E^) to the inner membrane of *Escherichia coli* when envelope stress is low. Elevated envelope stress triggers RseA cleavage by the sequential action of two membrane proteases, DegS and RseP, releasing σ^E^ to activate an envelope stress reducing pathway. Revertants of a Δ*degP* Δ*bamB* strain, which fails to grow at 37°C due to high envelope stress, harbored mutations in the *rseA* and *rpoE* genes. Null and missense *rseA* mutations constitutively hyper-activated the σ^E^ regulon and significantly reduced the major outer membrane protein (OMP) levels. In contrast, a novel *rpoE* allele, *rpoE3*, resulting from the partial duplication of the *rpoE* gene, increased σ^E^ levels greater than that seen in the *rseA* mutant background but did not reduce OMP levels. A σ^E^-dependent RybB::LacZ construct showed only a weak activation of the σ^E^ pathway by *rpoE3*. Despite this, *rpoE3* fully reversed the growth and envelope vesiculation phenotypes of Δ*degP*. Interestingly, *rpoE3* also brought down the modestly activated Cpx envelope stress pathway in the Δ*degP* strain to the wild type level, showing the complementary nature of the σ^E^ and Cpx pathways. Through employing a labile mutant periplasmic protein, AcrA_L222Q_, it was determined that the *rpoE3* mutation overcomes the Δ*degP* phenotypes, in part, by activating a σ^E^-dependent proteolytic pathway. Our data suggest that a reduction in the OMP levels is not intrinsic to the σ^E^-mediated mechanism of lowering envelope stress. They also suggest that under extreme envelope stress, a tight homeostasis loop between RseA and σ^E^ may partly be responsible for cell death, and this loop can be broken by mutations that either lower RseA activity or increase σ^E^ levels.

## Introduction

DegP is the major periplasmic protease in *Escherichia coli* [1]–[2]. Its proteolytic activity towards outer membrane proteins (OMPs) was initially observed against a mutant LamB protein with a temperature sensitive folding defect [3]. Subsequent studies showed an absolute or conditional requirement for DegP in cells either expressing folding-defective OMPs [4]–[5] or lacking a factor involved in OMP assembly [6]–[9].

Spiess *et al*. [10] first demonstrated that DegP possesses both protease and chaperone activities. A series of structural and biochemical studies on DegP provided important mechanistic clues [11]–[14]. These studies showed that the binding of unfolded substrate proteins to DegP triggers its reversible oligomerization into a cage-like structure and membrane association greatly influences DegP's assembly and activity. A full rescue of the temperature sensitive growth phenotype of Δ*degP* requires DegP's protease activity [10]. The expression of *degP* is under the control of the σ^E^ and Cpx regulons [15]–[18], which together constitute the two major envelope stress response pathways [19]–[21]. Both pathways are required to elevate *degP* expression to overcome the potentially lethal envelope stress caused by aberrant OMP assembly [9].

Compensatory mutations have been isolated that overcome the temperature sensitive growth phenotype of cells lacking DegP. The first of this was isolated by Baird and Georgopoulos [22] by selecting for revertants that grew at 42°C. One of the revertants displayed a cold sensitive growth phenotype and was found to affect a gene named *sohA* (suppressor of *htrA*) [22]. The mechanism by which *sohA* reversed the growth phenotype of Δ*degP* could not be determined. Prior to this study, Kiino and Silhavy [23] identified suppressor mutations of a LamB::LacZ hybrid protein in a locus they termed *prlF* and hypothesized that it was involved in some aspect of protein localization. The *prlF* gene, which turned out to be the site of the *sohA* mutation, together with *yhaV*, was recently determined to constitute a toxin-antitoxin module [24]. However, despite the new finding, the mechanism by which the *sohA*/*prlF* alleles overcome the growth defects of Δ*degP* remains unknown.

The second Δ*degP* suppressor mutation was obtained somewhat serendipitously and mapped to the *cyaA* gene [25]. It was concluded that the lower cAMP levels in the mutant *cyaA* Δ*degP* background overcame the temperature sensitive growth phenotype by inducing the σ^E^ and Cpx stress regulons. While it remains to be determined exactly how reduced cAMP levels leads to up-regulation of the envelope stress response systems, another alarmone, ppGpp, was shown to up-regulate σ^E^ activity in a RseA-independent manner [26]. In a follow-up study, the authors concluded that ppGpp affects σ^E^ activity both directly, by influencing σ^E^-dependent transcription, and indirectly, via altering competition for the core polymerase in favor of σ^E^ [27]. From these studies, it is apparent that the metabolic state of the bacterial cytoplasm also influences the envelope stress response systems.

In this study, we sought revertants of a Δ*degP* strain also lacking *bamB*, a nonessential component of the BAM complex [28]–[29]. The simultaneous absence of *degP* and *bamB* confers a severe growth phenotype even at 30°C [8]. We reasoned that the absence of DegP in conjunction with defective BAM machinery would yield potentially new kinds of suppressors that could give a novel insight into the envelope stress response. Indeed, *envZ* was identified as one of the genes in which suppressor alterations improved the growth phenotype of the Δ*degP* Δ*bamB* strain [30]. EnvZ and OmpR constitute a two-component signal transduction system [31], whose activity is modulated by a newly identified inner membrane protein, MzrA [30], [32]. MzrA directly interacts with EnvZ and changes its enzymatic activities so as to elevate the steady-state levels of OmpR∼P. Consequently, overexpression of MzrA or certain alterations in EnvZ constitutively activate the EnvZ/OmpR regulon. The activated EnvZ/OmpR regulon directly or indirectly (via inducing the expression of small regulatory RNAs) inhibits the synthesis of several OMPs and reduces envelope stress [30], [33].

Here we report two new sites of suppressor mutations that overcome the growth defects of a Δ*degP* Δ*bamB* strain. Mutations in *rseA* constitutively activate the σ^E^ regulon and reduce envelope stress in part by lowering the major OMP levels. In contrast, an *rpoE* mutation, which causes a novel duplication/truncation of the *rpoE* gene, does not reverse the growth defect by lowering the major OMP levels; instead, it appears to activate a proteolytic pathway that partly compensates for the loss of DegP.

## Results

### Mutations that overcome the temperature sensitivity phenotype of Δ*degP*

One of the ways to understand the consequences of a conditional lethal mutation is by isolating and characterizing revertants that have acquired so-called suppressor mutations and overcome the harmful effects of the original mutation. We have previously shown that a mutant simultaneously lacking *degP* and *bamB* displays a synthetic and conditional lethal phenotype at or above 37°C and grows poorly even at 30°C [8]. Because of this phenotype, the double mutant provides an ideal background in which to isolate suppressor mutations that reverse the growth defect, presumably by improving the OMP assembly environment and/or reducing envelope stress.

**Figure 1:**
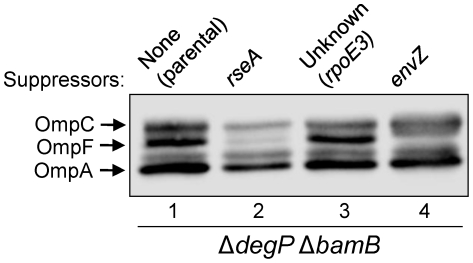
Profiles of major OMPs from the parental strain (Δ*degP* Δ*bamB*) and representative revertants carrying three different types of suppressor mutations. OMP levels were examined by Western blot analysis from overnight grown cultures at 30°C. Each lane contains protein samples from equal number of cells, based on OD_600_.

Liquid cultures of the Δ*degP* Δ*bamB* double mutant were grown in LB at 30°C, diluted, spread onto LB agar plates, and incubated at 37°C until colonies appeared (between 24 and 36 hours). Temperature resistant colonies, which arose at a frequency of around 10^−7^, were then purified at 30°C and suppressor mutants categorized based on growth robustness and OMP levels. Using these criteria, the suppressors were classified into three groups (Fig. 1). One group included suppressor mutations affecting *envZ* of the EnvZ/OmpR two-component regulon (Fig. 1, lane 4; [30]).

The second group of revertants were distinct from those carrying missense mutations in *envZ* in that the levels of all the major OMPs (Fig. 1, lane 2), not just that of OmpF as seen in the *envZ* mutants, were significantly reduced. Since this phenotype is reminiscent of an *rseA* null mutation, we determined the nucleotide sequence of the *rseAB-rpoE* region. Three different types of mutations were found in the *rseA* gene, resulting in (a) a W33R substitution, which was previously shown to directly affect the RseA-σ^E^ binding pocket [34], (b) a frame-shift mutation after the sixteenth codon of *rseA*, and (c) an IS*1* insertion element at nucleotide 454 of *rseA*. In each case, the mutations presumably abrogated RseA's ability to sequester σ^E^ at the inner membrane, thereby elevating the σ^E^-mediated envelope stress response. Thus, *rseA* mutations suppressed the Δ*degP* Δ*bamB*-mediated conditional lethal phenotype, in part, by lowering the load of OMPs in the envelope, and additionally by increasing the synthesis of periplasmic chaperones.

The last group was represented by one suppressor, which was noted to have the unusual ability to suppress lethality without significantly altering steady-state OMP levels (Fig. 1; lane 3). Through P1 transduction-mediated marker replacement, it was determined this third suppressor could fully correct the temperature sensitive growth defect of a Δ*degP bamB* ^+^ strain. Because of these two properties, we chose to investigate this suppressor further in the Δ*degP bamB* ^+^ strain and not in the original Δ*degP* Δ*bamB* strain, which grows very poorly and prone to accumulating suppressors.

**Figure 2:**
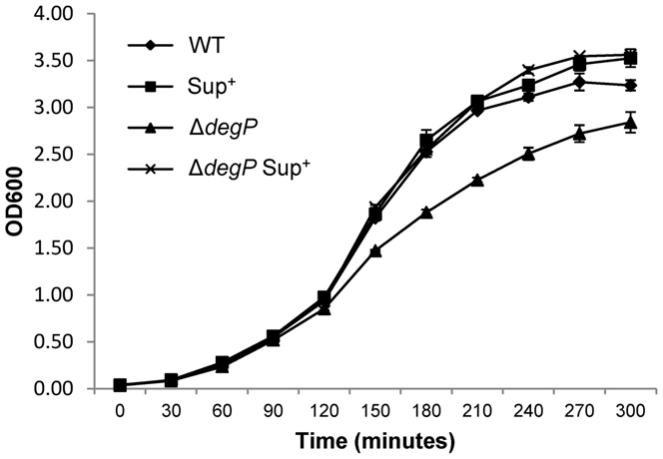
Growth curves of bacterial cultures grown at 39°C. Wild type and Δ*degP* cells, with or without the suppressor mutation, were grown overnight at 30°C. Next day, overnight cultures were diluted to 1∶100 in flasks containing fresh, pre-warmed, Luria broth and growth was resumed at 30°C and 39°C. OD_600_ was measured from bacterial samples withdrawn every 30 minutes. Only 39°C growth curves, obtained from two independent experiments, are shown. All strains grew almost identically at 30°C.

**Figure 3:**
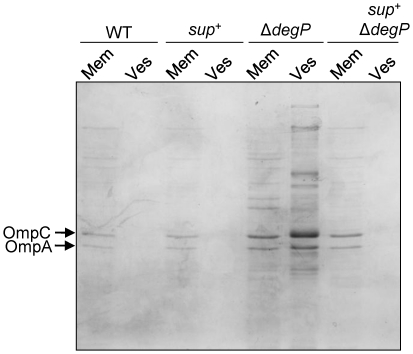
The suppressor mutation reverses the vesiculation phenotype of a Δ*degP* strain. Cultures were grown at 39°C in Luria broth for five hours, after which membranes and vesicles were prepared as described in the Experimental Procedure section. Samples were analyzed by SDS-PAGE and proteins were visualized after Coomassie blue staining. Abbreviations: mem, membrane; ves, vesicle; *sup* ^+^, the unknown suppressor mutation.

To quantify the extent to which the third suppressor mutation reversed the growth defect in Δ*degP* cells, growth was measured by culturing cells in LB for five hours at 39°C, which is the sub-lethal growth temperature for the Δ*degP* mutant, with cell density measured every thirty minutes. In contrast to Δ*degP*-only cells, Δ*degP* cells containing the suppressor mutation grew at a rate indistinguishable from the *degP* ^+^ strain (Fig. 2). *degP* ^+^ cells containing the suppressor mutation grew just like the *degP* ^+^ parental strain (Fig. 2). It is known that Δ*degP* cells vesiculate profusely [35]. We asked whether the suppressor mutation can reverse this phenotype of Δ*degP*. Membranes and vesicles obtained from cultures grown at 39°C were analyzed by SDS-PAGE and proteins were visualized after Coomassie blue staining (Fig. 3). As expected, Δ*degP* cells released a large amount of vesicles containing a variety of proteins, including OmpC and OmpA. However, the presence of the suppressor mutation almost completely reversed this phenotype of Δ*degP*. Hardly any proteins were visible from the vesicle fraction of wild-type and suppressor-containing cells (Fig. 3).

### Identification of the suppressor mutation

Before determining the mechanism of Δ*degP* suppression, we set out to identify the third suppressor mutation. For this, we introduced by P1 transduction a random Tn*10* (Tet^r^) insertion library into a Δ*degP* strain harboring the suppressor mutation and selected for tetracycline resistant (Tet^R^) transductants. Over a thousand Tet^R^ transductants were screened for the reversion to temperature sensitivity (i.e., loss of suppressor) by replica plating at 40°C and 30°C. One colony became temperature sensitive, indicating that either the insertion of Tn*10* in a gene confers the growth defect or the Tn*10* brought in the flanking wild type DNA and replaced the suppressor mutation. To distinguish the two possibilities, we determined the linkage of Tn*10* to the temperature sensitivity phenotype by transducing the Tet^R^ marker into the Δ*degP* strain containing the suppressor mutation. Approximately 50% of the time the resulting Tet^R^ transductants became temperature sensitive, indicating that the Tn*10* is closely linked to the suppressor mutation and not inserted in a gene that confers a temperature sensitive growth phenotype.

To conclusively determine the chromosomal location of the Tn*10*, arbitrarily primed polymerase chain reaction (AP-PCR) was utilized. PCR products obtained after the second, high-stringency reaction were analyzed on a 4% w/v agarose gel, excised and sequenced using the appropriate Tn*10* specific primers used in the reaction. Using this method, the Tn*10* was found to disrupt *yfiF* located at 58.5′ on the chromosome, at nucleotide 883. A known Kan^r^ insertion in *glrK* (57.9′ on the *E. coli* chromosome) was utilized to determine which side of *yfiF*::Tn*10* the suppressor mutation was located. Δ*glrK*::Kan^r^ was introduced into Δ*degP* suppressor-*yfiF*::Tn*10* cells by P1 transduction, and Kan^r^ transductants were screened for Tet^s^ and temperature sensitive phenotypes. Using this method, the gene order was determined to be *glrK*-suppressor-*yfiF*.

### Identification of the gene affected by the suppressor mutation

Several sets of primers were designed to amplify 1 to 3 kb long DNA from the 57.93 to 58.53 minute region of the chromosome from wild-type and suppressor-containing strains and the products were sequenced. When amplifying the *rpoE*-*nadB* region, we noted the presence of a PCR product from the suppressor strain that was approximately 1 kb larger than that amplified from the wild-type strain (data not shown). Sequence analysis indicated the presence of duplicated DNA in the suppressor strain.

**Figure 4:**
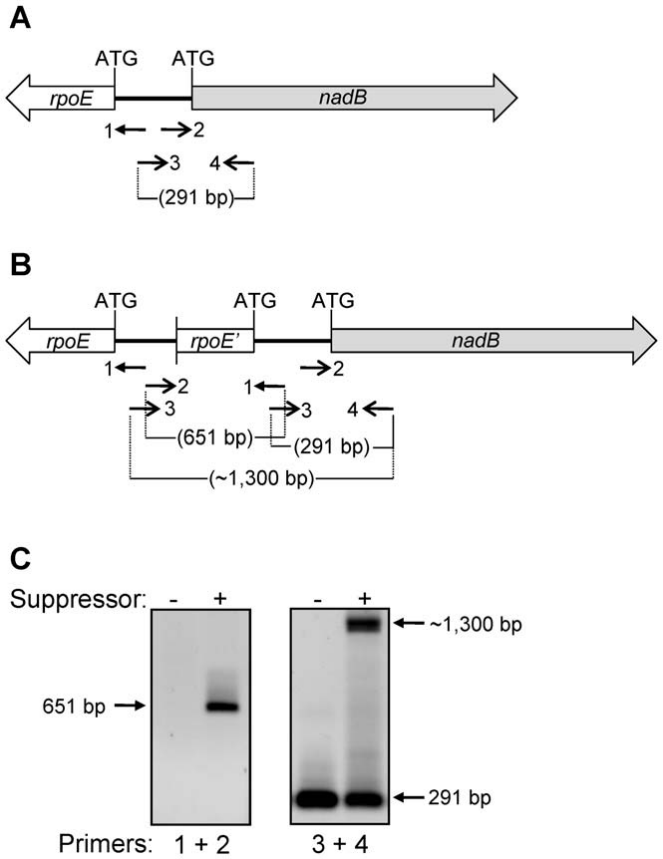
Identification of the genetic rearrangement in the suppressor strain. PCR amplifications were carried out to narrow down the site of possible genetic rearrangement in the *rpoE*-*nadB* region of the chromosome. (**A**, **B**) Approximate drawings showing the *rpoE* and *nadB* genes from the wild-type (**A**) and suppressor (**B**) strains, as well as the positions of primers (numbered 1 to 4), their orientations, and size of the amplified DNA fragments. (**C**) Agarose gels showing the results of PCR amplifications from the wild-type and suppressor strains. Numbers 1 to 4 refer to the primers shown in (**A**) and (**B**).

In order to conclusively determine the molecular nature of the suppressor mutation, a primer set was designed which consisted of one primer landing upstream of *rpoE* and reading into *rpoE* (primer 1) and one primer landing upstream of *nadB* and reading into *nadB* (primer 2) (Fig. 4A). Because *rpoE* and *nadB* are divergently transcribed, the only way to obtain PCR product is if there was truly a duplication of *rpoE* or *nadB,* such that the two “forward” primers were able to prime the reaction by facing each other in the chromosome. Indeed, when PCR was performed in the wild-type and suppressor strains, only the suppressor strain gave rise to a fragment using the divergent primer set (Fig. 4C). Another set of primers that points towards each other and lands 157 bp upstream of *nadB* ATG (primer 3) and 124 bp downstream from the *nadB* ATG (primer 4) produced an expected 291-bp long PCR fragment from wild type strain (Fig. 4C). However, the suppressor containing strain yielded two fragments of 291 bp and about 1.3 kb (Fig. 4C), confirming the presence of duplicated DNA (Fig. 4B).

The purified 651-bp long PCR product was sequenced using the same primers used for amplification, revealing a novel duplication-truncation of *rpoE* (Fig. 4B). The mutation consisted of *rpoE* truncated at nucleotide 396, corresponding to amino acid F122. After F122, the amino acid sequence continued with the non-native MVWYA sequence before reaching a stop codon (UAG). In addition to coding for five non-native amino acids, the region immediately after F122 was identical to the DNA sequence from 21 bases upstream of the *nadB* translation start to the translation start site of the native, full-length copy of *rpoE* was reached (Fig. 4B). Thus, the native copy of *rpoE* gene, present downstream of the 3′-tructaed copy of *rpoE*, is likely transcribed by the native promoter as well as the truncated *rpoE* gene promoter. We refer to the suppressor mutation as *rpoE3*, named after the suppressor isolate numbered 3.

### RpoE (σ^E^) levels are elevated in the rpoE3 background

**Figure 5:**
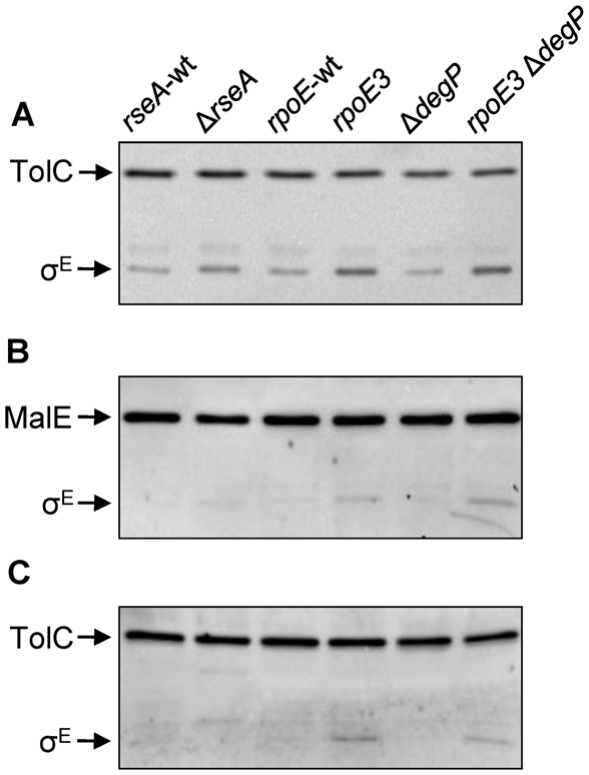
Examination of the σ^E^ (RpoE) levels from different genetic backgrounds and cell fractions. σ^E^ was detected by Western blots from protein samples obtained from whole cells (**A**), soluble (**B**, periplasm and cytoplasm) and insoluble (**C**, membranes) fractions. TolC-specific and σ^E^-specific antibodies were used in (**A**), while antibodies raised against σ^E^ and a MalE-TolC fusion protein were used in (**B**) and (**C**). The MalE-TolC fusion antibodies were used to verify the purity of soluble (MalE) and insoluble or membrane (TolC) fractions. Relevant genotypes of the strains are shown at the top.

Because of the partial duplication of *rpoE* in *rpoE3*, we asked whether the steady-state σ^E^ levels were elevated in cells harboring *rpoE3*. Wild-type and Δ*degP* cells containing *rpoE3* were grown to late exponential phase, and whole-cell extracts were isolated to analyze σ^E^ levels by Western blot using σ^E^-specific antibodies (Fig. 5). In strains containing the *rpoE3* allele, σ^E^ levels were four-fold (in *degP* ^+^ background) to six-fold (in Δ*degP* background) higher than in their *rpoE* ^+^ counterparts (Fig. 5A). This is likely due to elevated *rpoE* expression resulting from the *rpoE3*-mediated promoter duplication (in *degP* ^+^ background) and increased envelope stress (in Δ*degP* background). Despite these increases in σ^E^ levels, the steady-state levels of OMPs were not reduced in the *rpoE3* mutant (Fig. 1). In comparison, the σ^E^ level in a *rseA* null strain went up by 2.5 fold (Fig. 5A), which is lower than that seen in the *rpoE3* strain, yet OMP levels were drastically reduced in the *rseA* mutant background (Fig. 1). It is noteworthy that despite the existence of a truncated *rpoE* open reading frame in addition to the full length open reading frame, no small molecular weight bands reacted with the σ^E^-specific antibodies. The reason for this could be that either the antibodies used did not recognize the truncated σ^E^ protein, the truncated protein was not produced, or it was highly unstable.

σ^E^ must be released into the cytoplasm in order to become active. One possible explanation for the increase in σ^E^ levels without a concomitant reduction in OMPs in the *rpoE3* mutant is that, since *rseA* itself is a member of the σ^E^ regulon, the *rpoE3* mutation increases the level of membrane-bound σ^E^ rather than the soluble σ^E^, thus limiting the σ^E^ response. To test this, cells were grown at 37°C to late exponential phase, lysed by French press, and soluble and insoluble fractions were separated by centrifugation at 100 000 *g* for an hour. σ^E^ from the two fractions were analyzed by Western blot using anti-σ^E^ antibodies (Fig. 5B and C). Purity of the two fractions was affirmed by probing for TolC (insoluble) and MalE (soluble) proteins. Surprisingly, σ^E^ levels increased in both fractions (Fig. 5B and C). Quantification revealed that soluble σ^E^ levels increased 1.38 fold (Fig. 5B) and insoluble σ^E^ levels decreased 0.78 fold (Fig. 5C) in the *rpoE3* Δ*degP* strain compared to the *rpoE3* strain. Thus, despite the increase of σ^E^ in both fractions, there appears to be a small but preferential increase in the soluble active σ^E^ form in the *rpoE3* Δ*degP* strain. This is expected, since additional envelope stress caused by the loss of DegP would trigger a further release of σ^E^ from the envelope to combat stress.

### *rpo3* triggers a modest σ^E^ response

Based on the results obtained above, we hypothesized that the suppressor mutation exerted its effect by slightly activating the σ^E^ response without reducing the steady-state OMP levels (Fig. 1). To test this, *rpoE3* was introduced into cells containing a RybB::LacZ fusion. RybB, whose expression is controlled by σ^E^, encodes a small regulatory RNA responsible for down-regulating synthesis of several OMPs, including OmpC, OmpF and OmpA [36]–[38].

**Figure 6:**
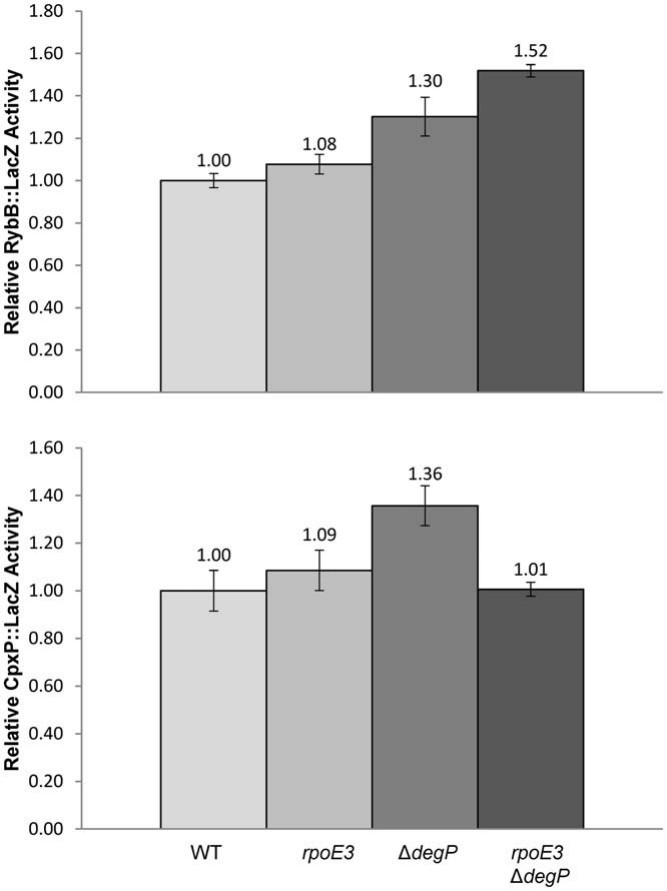
Effects of Δ*degP* and *rpoE3* mutations on RybB::LacZ (A) and CpxP::LacZ (B) activities. For each strain, two independent cultures were grown at 39°C to a mid-log phase and used for β-galactosidase assays. LacZ activities are relative to the wild-type strain. The relevant genotypes of the strains are labeled at the bottom.

RybB::LacZ activity was determined from wild type, *rpoE3,* Δ*degP* and *rpoE3* Δ*degP* cells grown at 39°C to mid- to late-exponential phase (Fig. 6A). In cells harboring the *rpoE3* allele, RybB::LacZ activity increased by about 10% relative to wild type, indicating only a slight activation of the σ^E^ response at this growth temperature. The Δ*degP* cells, which grew slightly slower than the parental strain (Fig. 2), showed a 30% increase in the RybB::LacZ compared to the parental strain. In the *rpoE3* Δ*degP* double mutant, which does not show a growth defect, an additive effect was observed: RybB::LacZ activity increased by 50% over wild type. This indicates that the two mutations elevated *rpoE* expression by independent mechanisms: *rpoE3* directly increased σ^E^ levels, while the Δ*degP* mutation indirectly activated σ^E^ by causing envelope stress. Only a modest increase in RybB::LacZ expression would help explain why there was no significant reduction in steady-state OMP levels in the *rpoE3* Δ*degP* background compared to the parental *rpoE* ^+^ *degP* ^+^ strain (Fig. 1). In contrast, the RybB::LacZ activity in an *rseA* null background went up almost six-fold (Gerken and Misra, unpublished data). This increase correlates well with a significant reduction in the OMP levels (Fig. 1). We also measured RybB::LacZ activity from cells grown at 37°C since there is a possibility that the relatively small difference observed between wild type and mutant strains grown at 39°C could be due to the basal RybB::LacZ activity in wild type strain being already high at 39°C. The results from 37°C growth experiments were similar to those obtained from cells grown at 39°C, with a relative increase of 20% (*rpoE3*), 20% (Δ*degP*), and 70% (*rpoE3* Δ*degP*) (Fig. S1). The additive effect of *rpoE3* and Δ*degP* was once again observed, indicating the two mutations activate RybB::LacZ expression by independent mechanisms.

### *rpo3* restores envelope homeostasis by lowering Δ*degP*-mediated activation of the Cpx pathway

Both Cpx and σ^E^ pathways are important in reducing envelope stress caused by aberrant β-barrel OMP assembly [9]. The Cpx-mediated upregulation of *degP* is critical for this reduction in envelope stress [9]. Consistent with this view, expression of *cpxP*, a prototypical member of the Cpx regulon, is modestly upregulated in Δ*degP* cells grown at 39°C (Fig. 6B; [9]). We asked whether the elevated σ^E^ response by *rpoE3* will help reduce the activated Cpx response observed in Δ*degP* cells. As shown in Fig. 6B, the Δ*degP*-mediated elevated CpxP::LacZ activity returned to the wild type level in the *rpoE3* Δ*degP* double mutant background. These results showed the complementary nature of the two envelope stress response pathways controlled by the σ^E^ and Cpx systems.

### Potential activation of a DegP-independent proteolysis pathway in the *rpoE3* mutant

One of the major functions of the σ^E^ response is to promote the proper folding and/or destruction of aberrantly folded OMPs in the envelope. In a Δ*degP* background, *rpoE3* obviously cannot simply increase *degP* transcription to compensate for increased stress and abolish the temperature sensitive growth phenotype. We therefore asked whether *rpoE3* caused the activation of another proteolytic pathway, thus allowing for normal growth in a Δ*degP* background.

For this, we employed AcrA_L222Q_, an unstable variant of AcrA that is rapidly degraded in stationary phase grown cultures at 37°C, but not at 30°C, in a DegP-dependent manner [39]. Because of its instability properties, AcrA_L222Q_ makes an ideal substrate to assay envelope proteolysis activity *in vivo*. The chromosomal allele of *acrA* expressing AcrA_L222Q_ was introduced into cells harboring *rpoE3* and lacking *tolC* (Δ*tolC* destabilizes the mutant AcrA protein). In order to test for proteolysis in this background, *degP* was also removed by a *degP*::Kan^r^ insertion.

**Figure 7:**
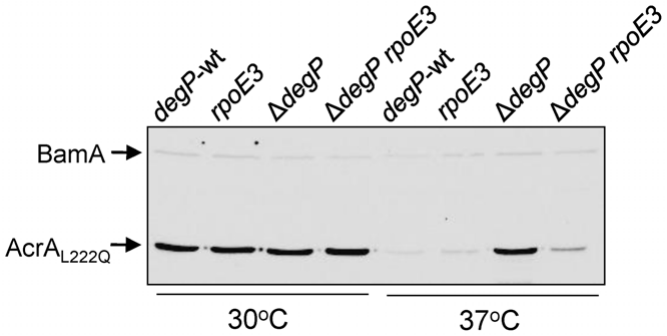
Levels of a labile AcrA variant, AcrA_L222Q_, in cells with various genetic backgrounds. Cultures were grown at 30°C and 37°C and AcrA_L222Q_ levels were determined from whole cell extracts by Western blot analysis. BamA, detected by BamA antibodies, was used as a gel loading control.

Cells were grown overnight in LB broth at 37°C and 30°C, after which samples were equalized according to final cell densities and boiled in SDS sample buffer. Samples were analyzed by Western blot using antibodies recognizing AcrA. At 30°C, the presence of *degP*::Kan^r^ or *rpoE3* did not significantly affect AcrA_L222Q_ levels (Fig. 7, lanes 1–4). However, at 37°C AcrA_L222Q_ levels increased six folds in a *degP*::Kan^r^ background compared to the *degP* ^+^ strain (Fig. 7, lanes 5 and 7). In an *rpoE3 degP* ^+^ background, the protein was also degraded due to the presence of intact DegP (Fig. 7, lane 6). Interestingly, the presence of *rpoE3* in the *degP*::Kan^r^ background caused a three-fold reduction in the AcrA_L222Q_ levels compared to that present in the *degP*::Kan^r^ background alone (Fig. 7, lane 8), reflecting decreased stability of the protein, presumably due to an increase in proteolysis in the envelope. Consistent with the involvement of a σ^E^-mediated, DegP-independent proteolytic pathway, AcrA_L222Q_ levels were also reduced in a Δ*rseA* Δ*degP* background where σ^E^ was fully activated (Fig. S2A).

*acrA* is not known to be a member of the σ^E^ regulon. Indeed, we have previously shown that the wild-type AcrA protein level did not change in a *bamA* mutant background in which the σ^E^ response was strongly induced [40]. Nevertheless, to eliminate the possibility that the expression of the *acrA* gene is somehow strongly affected by the induction of σ^E^ in the *rpoE3* background, we created an isogenic set of strains to those mentioned above, except that this set contained the wild type *acrA* gene. Western analysis data showed that at 37°C in the *rpoE3 degP*::Kan^r^ background, the level of wild type AcrA decreased only about 10% compared to the *rpoE* ^+^ Δ*degP* cells (Fig. S2B). In contrast, AcrA_L222Q_ levels were reduced over 300% in the *rpoE3 degP*::Kan^r^ background compared to *rpoE* ^+^ *degP*::Kan^r^ cells (Fig. 7). These data support the notion that a post-synthesis event, most likely an activated proteolytic pathway of the σ^E^ regulon, is primarily responsible for the observed destabilization/degradation of AcrA_L222Q_ in the absence of DegP. This pathway, in part, contributes to the mechanism of suppression by *rpoE3*.

## Discussion

One of the hallmarks of the fully activated σ^E^ system is the severely reduced major OMP levels resulting from high expression of the σ^E^-controlled small regulatory RNAs, RybB and MicA [36]–[38]. Indeed, *rseA* mutations that constitutively activate the σ^E^ stress response pathway also dramatically lower the major OMP levels (Fig. 1). Even though the *rpoE3* mutation significantly elevates σ^E^ in the cell—over two and fourfold compared to Δ*rseA* and *rpoE* ^+^ strains, respectively—no significant changes in the levels of major OMPs, OmpA and OmpC, were observed, indicating that the σ^E^ stress response pathway was not fully activated. This was also reflected by a mere 10% and 20% increase in σ^E^-controlled RybB::LacZ activity at 39°C and 37°C, respectively. We suspect that in the *rpoE3* background the continued presence of RseA at presumably slightly higher levels prevents σ^E^ from becoming fully active. In the *rpoE3* Δ*degP* background, the σ^E^ pathway is more active than in the *rpoE3* or *rpoE* ^+^ background, based on the elevated RybB::LacZ activity and a complete reversal of the vesiculation and temperature sensitive growth phenotypes of Δ*degP*. Yet, the absence of a significant effect on the major OMP levels suggests the extent of σ^E^ activation still remains lower in the *rpoE3* background than in a Δ*rseA* background.

In contrast to Δ*degP* cells grown at 39°C, a greater σ^E^ and Cpx activation is observed in cells with impaired OMP assembly pathways stemming from the loss of a major periplasmic chaperone, SurA, or the presence of a defective BAM complex [9], [40]. It is conceivable that differences in the biochemical nature of OMP assembly intermediates and their levels account for some of the disparities in envelope stress response in different genetic backgrounds. For example, it is well established that during aberrant OMP assembly, exposure of the C-terminal OMP residues in the periplasm activates the σ^E^ pathway by promoting RseA proteolysis [41]. This likely occurs in a background lacking SurA or expressing a defective BAM complex. In contrast, there is no evidence that OMP assembly is significantly defective in Δ*degP* cells, since we do not see any decrease in OmpA and OmpC levels from the envelopes. Nevertheless, it is likely that there is some increase in the nascent, unassembled OMPs that have fallen off the proper assembly pathway and are normally captured and/or degraded by DegP. Also, the levels of other unstable/unfolded envelope proteins, including those that normally reside in the periplasm, must increase in Δ*degP* cells grown at 39°C. Thus, envelope stress is likely to be created in Δ*degP* cells—enough to induce to a robust stress response through activating the σ^E^ or Cpx pathways. The predominant response to this stress appears to be the release of outer membrane vesicles, but this is clearly not sufficient when growth temperature reaches 40°C where viability of Δ*degP* cells becomes severely compromised. The fact that *rpoE3* completely reverses the phenotypes of Δ*degP* suggests that it must lower the levels of stress-causing envelope proteins. However, unlike the *rseA* null mutations, which significantly lower OMP levels, the effect of *rpoE3* on OMPs alone in not sufficient to account for its protective phenotype.

Since OMP levels are not reduced by *rpoE3*, alternative stress responses must account for the reversal of the Δ*degP* phenotypes. Our results indicate that up-regulation of a DegP-independent, σ^E^-dependent proteolytic pathway can partly accounts for this reversal. To test this pathway, we used a mutant AcrA protein, AcrA_L222Q_, which is rapidly degraded in a DegP-dependent manner [39]. In the absence of DegP, the mutant AcrA protein level rose significantly, but in the presence of *rpoE3*, AcrA_L222Q_ levels went down again. The level of AcrA_L222Q_ was also reduced in the absence of RseA, indicating the involvement of σ^E^. With no reduction in wild type AcrA levels in the *rpoE3* background, it appears that a post-synthesis step, most likely involving increased proteolysis, accounts for the reduced AcrA_L222Q_ level. We do not currently know the identity of the protease(s) responsible for AcrA_L222Q_ degradation in the absence of DegP in the *ropE3* and Δ*rseA* strains.

Overexpression of SohB and DegQ from plasmids has been reported to rescue the temperature sensitive growth phenotype of Δ*degP* [42]–[43]. However, neither *sohB* nor *degQ* is known to be regulated by σ^E^ and there is no experimental data showing SohB is actually a protease. *yfgC*, a gene regulated by σ^E^, is predicted to encode a periplasmic metalloprotease [44]. However, deletion or overexpression of YfgC did not influence AcrA_L222Q_ stability or the Δ*degP* phenotypes (data not shown). It should be noted that activation of a proteolytic pathway likely represents only a component of the broader defensive response triggered by σ^E^. Therefore, we believe that the collective action of increased proteolysis and other defensive measures most likely account for the *rpoE3*-mediated reversal of the Δ*degP* phenotypes.

The fact that mutations required to increase σ^E^ levels and rescue the Δ*degP* phenotypes are either in *rseA*, a gene encoding for the negative regulator of σ^E^, or the *rpoE* gene itself suggests that even under high envelope stress conditions the amount of free σ^E^ becomes limiting. If growth at high temperatures without DegP indeed leads to the accumulation of misfolded OMPs, then these accumulated OMPs should trigger the DegS/RseP-mediated proteolytic pathway to degrade RseA and elevate free σ^E^ levels [45]. The fact that Δ*degP* cells begin to die at 39°C suggests that the defensive response system may have maxed out, perhaps because newly released and activated σ^E^ allows for a greater synthesis of RseA, which in turn re-captures σ^E^. In other words, under extreme envelope stress, a tight homeostasis loop between RseA and σ^E^ may partly be responsible for cell death. This loop can be broken by mutations that either lower RseA activity or increase σ^E^ levels. An alternative means to overcoming the growth phenotype of Δ*degP* without directly influencing the RseA-σ^E^ loop would be through mutations that influence the cAMP-CRP [25] or EnvZ/OmpR [30] pathway. The *prlF*/*sohA* mutations likely provide yet another mechanism to overcome the temperature sensitive growth phenotype of Δ*degP*.

## Materials and Methods

### Bacterial strains, media and reagents

*Escherichia coli* K-12 bacterial strains used in this study were constructed in an MC4100 background [46] and are shown in the Table S1. Luria-Bertani broth (LB) and Luria-Bertani agar (LBA) were prepared as previously described [47]. When required, media were supplemented with 50 µg ml^−1^ ampicillin, 25 µg ml^−1^ kanamycin, 12.5 µg ml^−1^ chloramphenicol, 10 µg ml^−1^ tetracycline and 0.2% L-(+) arabinose (Sigma). All other chemicals were of analytical grade. Bacterial growth curves were performed by diluting overnight cultures 1∶100 into 25 ml fresh pre-warmed media supplemented with appropriate antibiotics. Cultures were vigorously shaken at 39°C in baffle-bottom flasks in a water incubator with cell density checked every 30 min.

### DNA manipulation

Standard bacterial genetic methods were carried out as previously described [47]. Chromosomal deletion of d*egP*, followed by the removal of the antibiotic resistance cassette was carried out using the λ-red mediated gene deletion method as previously described [48]. Primer sequences are available upon request.

The unknown Tn*10* marker was identified by the arbitrary-primed PCR (AP-PCR) method [49]–[50]. The first, low-stringency round of PCR was carried out with wild-type cells and cells containing the unknown Tn*10* marker (see below). That round of PCR used both ARB1 and ARB6 primers [50], as well as either Tn*10* left or Tn*10* right primers to amplify fragments on either side of the Tn*10*. First round products were analyzed on an 0.8% agarose gel, and fragments unique to the Tn*10* strain were excised using a QIAquick gel extraction kit (QIAGEN) and subjected to a second, high-stringency round of PCR using the appropriate Tn*10* primer and ARB2 [50]. Amplified products were cleaned up using a QIAquick PCR purification kit and sequenced using the appropriate Tn*10* primer or ARB2. Sequences were analyzed to obtain non-Tn*10* sequence, and unassigned sequences were analyzed using BLASTn to determine the location of the Tn*10*.

### Cell fractionation

Fresh cultures were grown by sub-culturing overnight cultures into fresh media with appropriate antibiotics at a starting OD_600_ of 0.025. Cell pellets, collected from freshly grown cells, were resuspended in a lysis buffer containing 20 mM Tris-HCl pH 7.5, 2 mM phenylmethylsulfonylfluoride (PMSF), 10 mM MgCl_2_ and 25 µg ml^−1^ DNase I. Samples were passed twice through a French pressure cell, followed by low-speed centrifugation of lysates to remove unlysed cells. Crude lysates were the centrifuged at 100,000 *g* for 1 h at 4°C to pellet membranes. Periplasm was extracted using the gentle osmotic shock method [51]. Prior to French Press lysis, cell pellets were resuspended in periplasm extraction buffer (10 mM Tris-HCl pH 7.5, 500 mM sucrose, 10 mM EDTA and 0.2 mg ml^−1^ lysozyme) and incubated on ice for 30 min. Samples were centrifuged at 100,000 *g* for 15 min and periplasm drawn off as soluble fraction. Spheroplast pellets were then washed with cold 10 mM Tris-HCl pH 7.5 and subjected to French press lysis as described above. After high speed spin, cytoplasm was harvested from the soluble fraction. Membranes pellets were routinely washed in 10 mM Tris-HCl pH 7.5 and resuspended according to cell density in the same buffer.

### Protein methods

For Western blot analysis from whole cells, aliquots of cultures, normalized to cell density, were pelleted in a microcentrifuge. Pellets were resuspended in sample buffer (62.5 mM Tris-HCl pH 6.8, 10% v/v glycerol, 100 µg ml^−1^ bromophenol blue, 5% v/v β-mercaptoethanol and 2% w/v sodium dodecyl sulfate [SDS]). Samples were boiled for 5 min and analyzed by SDS-polyacrylamide gel electrophoresis (SDS-PAGE). When required, 4 M urea was added in order to resolve OmpC and OmpF. Membrane, periplasmic and cytoplasmic fractions were prepared in the same buffer as whole-cell samples. After electrophoresis, proteins were transferred to Immobilon-P polyvinylidene difluoride (PVDF) membrane (Millipore) using a Bio-Rad mini-transblot. Membranes were blotted with antibodies raised against OmpF (1∶16 667), LamB (1∶10 000), AcrA (1∶10 000), TolC (1∶10 000), MalE (1∶10 000) RpoE (1∶5 000) or GroEL (1∶25 000). Blots were developed as described previously [40]. Protein bands were quantified using Bio-Rad Quantity One software. When appropriate, SDS-PAGE gels were stained with Coomassie brilliant blue R-250 (Sigma).

### Enzymatic assays

β-galactosidase activity was determined by an established method [52], modified for use with microtiter plates. Kinetic analysis of β-galactosidase activity was carried out using a Versamax microtiter plate reader (Molecular Dynamics). Activity was calculated as the rate of ONPG (ortho-Nitophenyl-β-D-galactopyranoside) cleavage (OD_420_) normalized to cell density (OD_562_) in each well. All assays were performed in quadruplicate.

## Supporting Information

Figure S1 **Effects of Δ** ***degP*** **and** ***rpoE3*** **mutations on RybB::LacZ activity.** Two independent cultures were grown at 37°C to a mid-log phase and used for β-galactosidase assays. LacZ activities are relative to the wild-type strain. Relevant genotypes are shown at the bottom.
(TIF)

Figure S2 **Levels of AcrA_L222Q_ (A) and wild type** ***acrA*** **(B) in different genetic backgrounds.** AcrA levels were determined by Western blot analysis of whole cell lysates prepared from overnight grown cultures at 37°C. GroEL, LamB and AcrA were detected using specific antibodies. GroEL served as a gel loading control. Each lane contains protein samples from equal number of cells, based on OD_600_. Relevant genotypes are shown at the top.
(TIF)

Table S1 **A list of** ***Escherichia coli*** **K-12 strains used in this study.**
(DOC)
