# Supplementary figures and images for: Reversal of the ΔdegP Phenotypes by a Novel rpoE Allele of Escherichia coli
Source: PLoS One. 2012 Mar 16;7(3):e33979. doi: 10.1371/journal.pone.0033979 (PMC3306311; doi:10.1371/journal.pone.0033979)

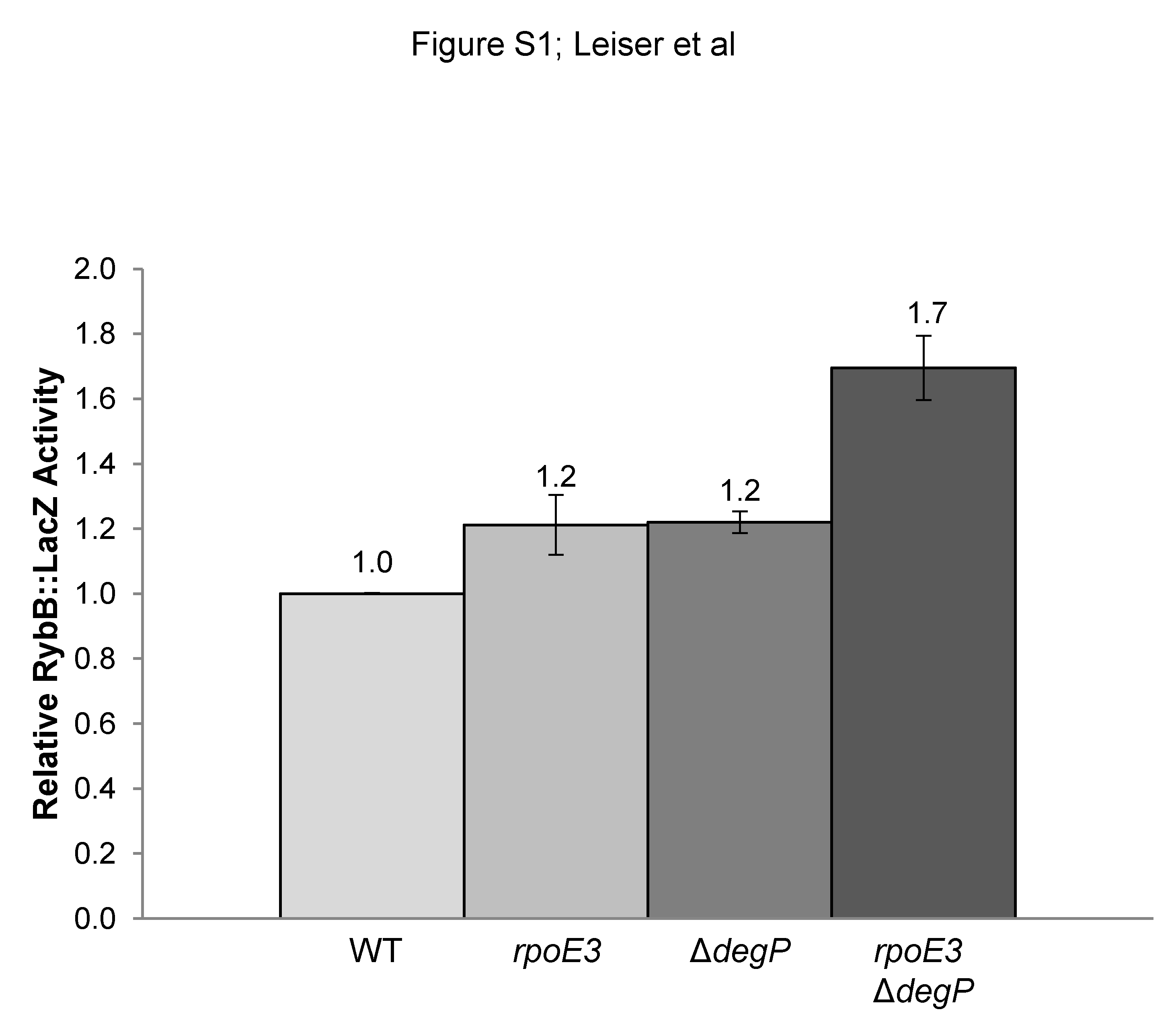

Supplement: Figure S1 — Effects of Δ degP and rpoE3 mutations on RybB::LacZ activity. Two independent cultures were grown at 37°C to a mid-log phase and used for β-galactosidase assays. LacZ activities are relative to the wild-type strain. Relevant genotypes are shown at the bottom. (TIF) [file pone.0033979.s001.tif]

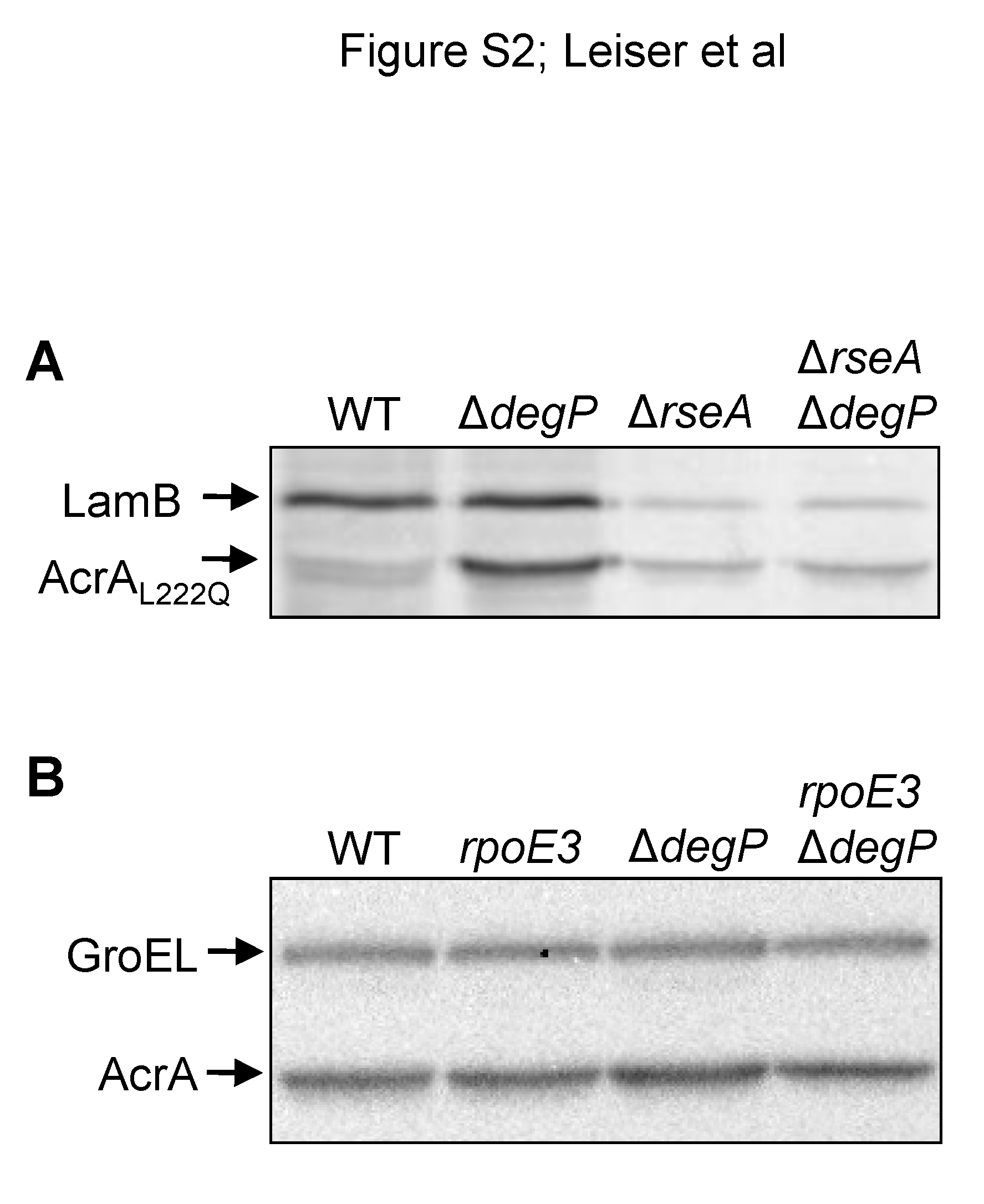

Supplement: Figure S2 — Levels of AcrAL222Q (A) and wild type acrA (B) in different genetic backgrounds. AcrA levels were determined by Western blot analysis of whole cell lysates prepared from overnight grown cultures at 37°C. GroEL, LamB and AcrA were detected using specific antibodies. GroEL served as a gel loading control. Each lane contains protein samples from equal number of cells, based on OD600. Relevant genotypes are shown at the top. (TIF) [file pone.0033979.s002.tif]
