# Supplementary material for: Reversal of the ΔdegP Phenotypes by a Novel rpoE Allele of Escherichia coli
Source: PLoS One. 2012 Mar 16;7(3):e33979. doi: 10.1371/journal.pone.0033979 (PMC3306311; doi:10.1371/journal.pone.0033979)
Supplement: Table S1 — A list of Escherichia coli K-12 strains used in this study. (DOC) [file pone.0033979.s003.doc]

**Table S1.** *Escherichia* *coli* K-12 strains used in this study.

| Strains | Characteristics | Source |
| --- | --- | --- |
| MC4100 | F- *araD139* Δ(*argF*-*lac*)U139 *rspL150* *relA1* *flbB5301* *ptsF25* *deoC1* *thi-1* *rbsR* | [46] |
| RAM1318 | MC4100 Δ*ara714* Δ*bamB*::scar *degP*::Tn*10* | [8] |
| RAM1541 | MC4100 Δ*ara714* *envZ* [R397L] | [30] |
| RAM2011 | MC4100 Δ*ara714* Δ*degP*::scar | This study |
| RAM2012 | MC4100 Δ*ara714* Δ*degP*::scar *yfiF*::Tn*10* | This study |
| RAM2013 | MC4100 Δ*ara714* Δ*degP*::scar *rpoE3* *yfiF*::Tn*10* | This study |
| RAM2014 | MC4100 Δ*ara714* *yfiF*::Tn*10* Δ*rseA*::Km^r^ | This study |
| RAM2015 | MC4100 Δ*ara714* Δ*degP*::scar *yfiF*::Tn*10* Δ*rseA*::Km^r^ | This study |
| RAM2016 | MC4100 Δ*ara714* Δ*tolC*::Cm^r^ Δ*tolC*::Cm^r^ *acrA*[L222Q] | This study |
| RAM2017 | MC4100 Δ*ara714* Δ*tolC*::Cm^r^ Δ*tolC*::Cm^r^ *acrA*[L222Q] *rpo3* | This study |
| RAM2018 | MC4100 Δ*ara714* Δ*tolC*::Cm^r^ Δ*tolC*::Cm^r^ *acrA*[L222Q] Δ*degP*::scar | This study |
| RAM2019 | MC4100 Δ*ara714* Δ*tolC*::Cm^r^ Δ*tolC*::Cm^r^ *acrA*[L222Q] Δ*degP*::scar *rpo3* | This study |
| RAM1978 | MC4100 Δ*ara714* Δ*tolC*::Cm^r^ Δ*tolC*::Cm^r^ *acrA*[WT] | This study |
| RAM1979 | MC4100 Δ*ara714* Δ*tolC*::Cm^r^ Δ*tolC*::Cm^r^ *acrA*[WT] *rpo3* | This study |
| RAM1980 | MC4100 Δ*ara714* Δ*tolC*::Cm^r^ Δ*tolC*::Cm^r^ *acrA*[WT] Δ*degP*::scar | This study |
| RAM1981 | MC4100 Δ*ara714* Δ*tolC*::Cm^r^ Δ*tolC*::Cm^r^ *acrA*[WT] Δ*degP*::scar *rpo3* | This study |
| RAM2020 | MC4100 Δ*ara714* Δ*rybB*::*lacZ* *yfiF*::Tn*10* | This study |
| RAM2021 | MC4100 Δ*ara714* Δ*rybB*::*lacZ* *rpoE3* *yfiF*::Tn*10* | This study |
| RAM2022 | MC4100 Δ*ara714* Δ*rybB*::*lacZ* Δ*degP*::Cm^r^ *yfiF*::Tn*10* | This study |
| RAM2023 | MC4100 Δ*ara714* Δ*rybB*::*lacZ* Δ*degP*::Cm^r^ *rpoE3* *yfiF*::Tn*10* | This study |
| RAM2024 | MC4100 Δ*ara714* Δ*cpxP*::*lacZ* *nadA*::Tn*10* Δ*rseC*::Km^r^ | This study |
| RAM2025 | MC4100 Δ*ara714* Δ*cpxP*::*lacZ* *nadA*::Tn*10* *rpoE3* Δ*rseC*::Km^r^ | This study |
| RAM2026 | MC4100 Δ*ara714* Δ*cpxP*::*lacZ* *nadA*::Tn*10* Δ*degP*::Cm^r^ Δ*rseC*::Km^r^ | This study |
| RAM2027 | MC4100 Δ*ara714* Δ*cpxP*::*lacZ* *nadA*::Tn*10* Δ*degP*::Cm^r^ *rpoE3* Δ*rseC*::Km^r^ | This study |
